# Automated Object Detection, Mapping, and Assessment of Roadside Clear Zones Using Lidar Data

**DOI:** 10.1177/03611981211029934

**Published:** 2021-08-31

**Authors:** Maged Gouda, Bruno Arantes de Achilles Mello, Karim El-Basyouny

**Affiliations:** 1 Graduate Research Assistant, Department of Civil and Environmental Engineering, University of Alberta, Edmonton, Canada; 2 Undergraduate Research Assistant, Department of Electrical and Computer Engineering, University of São Paulo, São Carlos, Brazil; 3 Associate Professor and City of Edmonton’s Urban Traffic Safety Research Chair, Department of Civil and Environmental Engineering, University of Alberta, Edmonton, Canada

## Abstract

This paper proposes a fully automated approach to map and assess roadside clearance parameters using mobile Light Detection and Ranging (lidar) data on rural highways. Compared with traditional manual surveying methods, lidar data could provide a more efficient and cost-effective source to extract roadside information. This study proposes a novel voxel-based raycasting approach focused primarily on automating roadside mapping and assessment. First, the scanning vehicle trajectory is extracted. Pavement surface points are then detected, and a method is proposed to extract pavement edge trajectories. Once pavement edges are extracted, guardrails were identified using a conical frustum emitted from the edge trajectory points. Target points and flexion points are then generated and located on the roadside, and a voxel-based raycasting approach is used to search for roadside obstacles and query their locations. Finally, roadside slopes and embankment heights were mapped at specific intervals, and roadside design guidelines and requirements were automatically checked against the mapping results. Noncompliant locations with substandard conditions were automatically queried. The method was tested on four highway segments in Alberta, Canada. The accuracy of the edge detection reached up to 98.5%. Furthermore, the method proved to be accurate in object detection, being able to detect all obstructions on the roadside in each tested segment. The proposed method can help transportation authorities automatically map and inventory roadside clearance parameters. Moreover, the safety performance of existing road infrastructure can be studied using collected information and crash data to support decision making on road maintenance and upgrades.

Roadway departure crashes have some of the highest death rates among vehicle accident types, estimated to account for more than half of traffic fatalities (*1*). One effective method to alleviate these incidents is the addition of large roadside clear zones. Roadside clear zones are defined as the traversable area directly adjacent to the edge of a roadway, including the shoulder (*2–6*). These areas allow errant vehicles an opportunity to stop or recover safely in the event of roadway departure. Clear zones of increased width correlate with a lower probability of collision for errant vehicles, with zones 9 m wide allowing sufficient space for vehicles to safely stop or recover 85% of the time (*7*). Collision risk can be further reduced by lowering the slope of the clear zone. Slopes below 1V:4H are considered recoverable, while steeper slopes do not permit the majority of vehicles to return to the roadway (*2–4*, *8*). Higher slopes also lead to increased rollover risk and loss of control as the vehicle loses contact with the ground (*9*). Maximum crash risk exists in areas of high object density positioned close to the side of the road (*10*).

Unfortunately, relocation of all obstacles is not always possible for reasons such as right-of-way limitations or cost (*4*, *11*). Alternative solutions exist to improve safety, such as the installation of roadside barriers (*2–4*). Barriers have been shown to almost always reduce the probability of injury compared with hitting a roadside obstacle at an equal distance from the travel lane (*12*). One exception to this is that barriers may themselves act as obstacles at their endpoints. Barriers should, therefore, be installed only in areas of high obstacle volume where removal of obstructions is not possible (*13*). To determine this, transportation agencies have traditionally needed to manually document roadside clear zone size and slope, and the position of obstructions on a case-by-case basis. Recently, however, because of its millimeter-level accuracy, reduction in cost, and widespread data collection capabilities, civil engineering research has shifted to the use of lidar as a fast and feasible alternative for data collection (*14–20*).

Lidar technology uses high-frequency laser scanners to map its surroundings by creating high-resolution 3-D point clouds (*19*, *21*, *22*). Additional data, such as scan angle and intensity of the returned light beam, are also recorded (*18*). A specific variant of this technology known as Mobile Laser Scanning (MLS) can be used for efficient recording of road and roadside data (*23*). MLS involves mounting lidar scanners on vehicles, allowing data collection to be done rapidly without disruption to traffic. The collection of large-scale roadside data using lidar also offers significant cost savings compared with manual surveying (*24*, *25*).

Several methods using lidar data have recently been proposed, which explore obstacle detection and road/lane edge detection. However, the majority of these methods disregard rural environments and assessments beyond the pavement surface (*26–29*). Our approach solves limitations in previous research as it provides a holistic approach to object detection, roadside clearance mapping, and assessment using MLS data, a topic that has not been explored in previous research. The target in most previous studies was the detection of a certain element of the road. For instance, several studies investigated the extraction of pavement surface points, while others attempted extraction of road edges. Other studies focused on detecting a certain feature, such as roadside trees on urban roads or buildings (*30*, *31*).

This paper presents an automated voxel-based raycasting method for collecting inventories of roadside assets and mapping and assessing roadside clearance parameters (e.g., distance from features to the road edge, grades, embankment heights, etc.) using MLS data. The data collected were used to determine compliance with design guidelines (*4*). The method proposed in this study can help transportation authorities automatically detect locations with substandard conditions compared with design guidelines on large road networks. The information collected can be mapped with road collisions in a geographic information system software to further study the relationship between crashes and existing roadside parameters. As such, it can help develop performance-based design guidelines for roadside clear zones and support decision making on road maintenance and upgrades.

## Literature Review

In 2015, Ural et al. (*30*) investigated roadside feature extraction with orthophotos and lidar data using a semi-automated approach. Initially, the road surface was extracted from an orthorectified image using Support Vector Machine (SVM) classifiers. Buildings were then identified and excluded to avoid them being classified as road because of their similar appearance in images. The road surface was then reconstructed using a series of morphological image processing techniques. Estimation was then done to determine road width and assign a 20 ft buffer zone on both sides. Roadside obstructions were then located within clear zone boundaries from aerial lidar data. This was done by filtering ground points and then identifying trees and buildings using a graph-cut optimization algorithm. Testing was done on 23.6 mi of rural roadways in Indiana, USA. Road identification rates reached 90%. For building identification, the method delineated 140 buildings, 43 of which were false positives (e.g., vegetation, parts of roads, bridges, etc.). Of 107 ground truth buildings, 97 were identified, of which seven had more than 50% of their area missing, four had between 30% and 50% missing, nine lost less than 30%. The accuracy of edge detection was not discussed in the study. Roadside slopes were extracted at preidentified cross-section locations using a low-resolution Digital Elevation Model (DEM). The study did not explore a full approach to mapping roadside parameters (e.g., distance to obstacles, embankment heights, etc.). The authors recommended investigating the use of mobile lidar in future research.

Cabo et al. (*26*) presented a method for the extraction of road edges from MLS point clouds in 2016. Scan data were analyzed as individual polylines. Each line was split into approximately linear sections and simplified with a 3-D Douglas Peucker algorithm. Parallel lines were then grouped, and adjacent line sections of similar angles were combined into planar surfaces. The road edge was identified as the boundary of on- and off-road planar surfaces and then smoothed. Testing was done on an 2.1 km stretch of road containing 85 million scan points. Correctness and completeness of the predicted road surface were 99.1% and 97.5%, respectively. The tested road had a very high point density. High point density is common for urban scanning using MLS because of, in part, the low speed of the vehicle. In addition, the existence of a curb line that defines the road edge makes the detection of edges a relatively easy process compared with rural roads.

Yadav et al. (*27*) proposed a method for the detection of road surfaces from MLS data. Point cloud data was first segmented into rectangular sections on the x–y plane. Ground points were then filtered out of each segment by extracting all points with z values below a specific height, estimated at a value of high point density within segments. This estimation was refined by removing vertically outlying points from the predicted surface layer. Filtering was then done based on the consistent intensity and high point density on the road surface. Additional filtering was then done to connect sections together and add road markings previously lost as a result of intensity filtering. Finally, reconstruction was done by fitting splines to road curb points to add road sections blocked by obstructions during the scan. The testing of the algorithm was done at three test sites in Bengaluru city with double-pass MLS. Road surface extraction was accurate, with average completeness and correctness of 93.8% and 98.3%, respectively.

In 2018, Gu et al. (*28*) investigated a method of reliable road edge extraction from sparse lidar point cloud data. Multiple consecutive frames of point clouds were combined and used to capture more details and object features. The method’s framework consisted of pre-processing the point cloud using ICP registration on multiple frames to create a dense point cloud. Ground plane segmentation was then done using Linefit and RANSAC. Finally, road edge points were extracted and boundaries generated.

In 2016, Qiu et al. (*29*) presented a fast and robust algorithm to extract road edges from lidar data. The algorithm takes advantage of the elevation difference at the edge of roads. Previous approaches have been primarily developed for urban road environments, but are inefficient, and are sensitive to specific parameters. This algorithm attempts to address these limitations. The method first partitions point clouds into cross sections based on the GPS time of the points, and then denoises them. The point clouds may contain too many points, which lowers performance. Non-uniform density was another issue that was addressed by down-sampling to a similar density. Candidate road edge points were extracted with RANSAC by detecting different planes of the surfaces. The points were then refined. The method was tested on highways, urban roads, rural roads, and national primary roads. The scenes contained many grass-soil edges of roads, curb and fence edges, cars parked on the sides of roads, trees, poles, and buildings, which all complicate the extraction process. Completeness and correctness were typically in the high 90% range, except for challenging cases where there were many obstructions by the roadside, or there was almost no height difference at the road edges.

In 2019, Chen et al. (*31*) developed a feasible workflow for urban tree inventory using 3-D point clouds acquired from MLS. Few cities have tree inventories because of the cost and limitations of traditional tree inventory methods. Trees provide many benefits, such as reducing noise but are also a safety concern. Tree inventories are important for dealing with these issues; for example, knowledge about tree species can identify places where breakable roadside trees can be planted, which reduce crash damage. Their method first extracted individual trees using either euclidean clustering, Ncut, or region growing segmentation, followed by a 2-D alpha shape method to obtain cluster outlines. Then, parameters such as tree height and diameter at breast height were estimated, and the tree species was classified. Finally, the estimations and results were validated. This study showed the feasibility of MLS in helping with roadside tree inventory; however, some problems remain unsolved. Outlier points were hard to remove. Because MLS data is very large, it needs to be partitioned and processed individually. One limitation of the work was that objects near the partition boundaries may be divided into parts, causing errors. This process was not completely automated as outlier removal may still require manual intervention.

Since our proposed method detects objects with voxel-based raycasting using terrestrial rather than aerial scanning, it captures any 3-D obstacle on the side of the road, rather than a classification of certain objects such as trees or buildings only. This includes other vertical obstruction detection such as poles (*30*, *31*). In Ural et al. (*30*), a Digital Surface Model (DSM) was used in filtering buildings. DSMs, if used in a raycasting technique, can produce several false positives underneath elevated objects because of their form as a Triangular Irregular Network (TIN) (*18*, *19*). DSMs are also limited in their ability to represent overlapping objects when projected to a 2-D plane (*18*, *19*). Therefore, DSMs are not adequate in accounting for vertical surfaces (elevation difference at the same horizontal location) and thus are considered a 2.5-D approach rather than 3-D (*32*).

As is evident from the review, previous studies did not explore or research automating the process of mapping roadside clearance parameters (e.g., distance from features to the road edge, grades, embankment heights, etc.). A complete safety assessment of the roadside environment requires detecting any 3-D object existing in such an area, regardless of size, type, shape, or physical characteristics. Additionally, the same could be said for the slope of the roadside. It must conform to maximum steepness guidelines; therefore, it should be included in the analysis at a high level of detail and in a segmented form. This is important as design guidelines’ desirable clear zones change depending on the sequence of existing slopes. For instance, a recoverable slope must be provided after a non-recoverable slope to give an errant vehicle a chance for recovery (*4*). Besides, mitigation measures and clear zone upgrades defined in design guidelines are based on collision experience observed at a site. The automatic mapping and assessment of roadside characteristics to the required extent have not yet been developed in research. Thus, in this study, we propose a novel automated approach for performing the roadside mapping and assessment process using MLS data.

## Data Collection

Lidar data collection was performed by Alberta Transportation in 2015 using Tetra Tech PSP-7000, a proprietary multifunction pavement surface profiling vehicle. The vehicle was equipped with a RIEGL VMX 450 system to collect 360° lidar point clouds on rural highways in the province of Alberta, Canada. Surveys were conducted in normal traffic flow at speeds of up to 100 km/h. Collected data for a given highway were saved in 4-km segments in separate LAS files, each around 500 MB. Surveys performed at 90 km/h generate point densities in the range of 150 and 1,000 points/m^2^. In this study, two segments on Highway 2, 4 km each, a 4-km segment on Highway 63, and a 4-km segment on Highway 35 were included.

### Test segments

**Figure 1.**
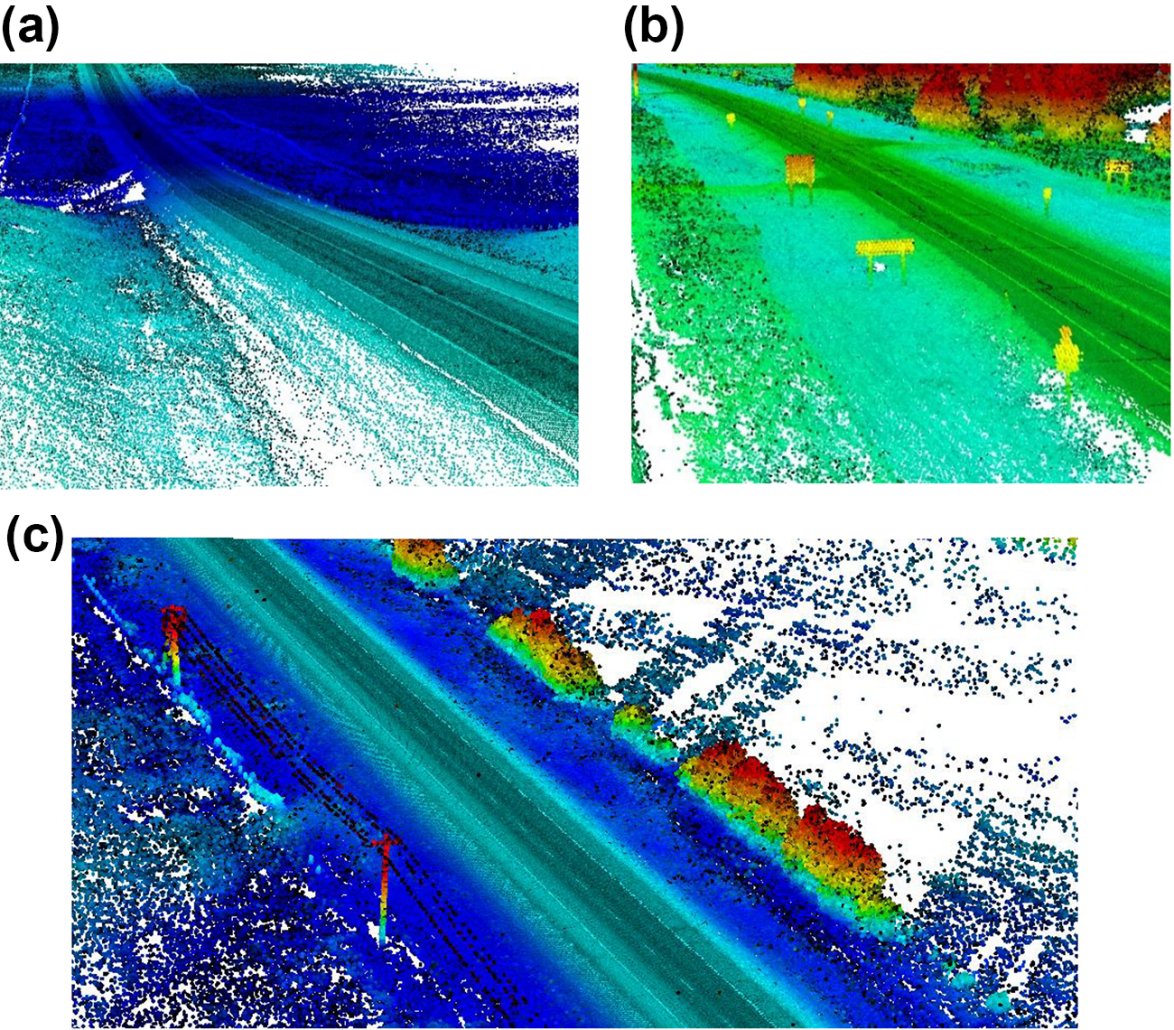
Collected point cloud data.

- Highway 2 – segment 1 and segment 2: two segments, 4 km each, from this highway were used for analysis (Figure 1*a* and *b*). The segments are from a two-way two-lane highway. Segment 1 starts at the Carway US–Canada border and extends 4 km to the north. Segment 2 is located approximately 300 km northwest of the city of Edmonton.
- Highway 63: a 4-km segment from this highway was used for analysis (Figure 1*c*). This is a two-way two-lane segment, located 90 km northeast of the city of Edmonton.
- Highway 35: a 4-km segment from this highway was used. The segment is a two-way two-lane road located 710 km northwest of the city of Edmonton.

Manual surveying data at the test segments was not available and, as such, other possible validation methods were used.

### Methodology

**Figure 2.**
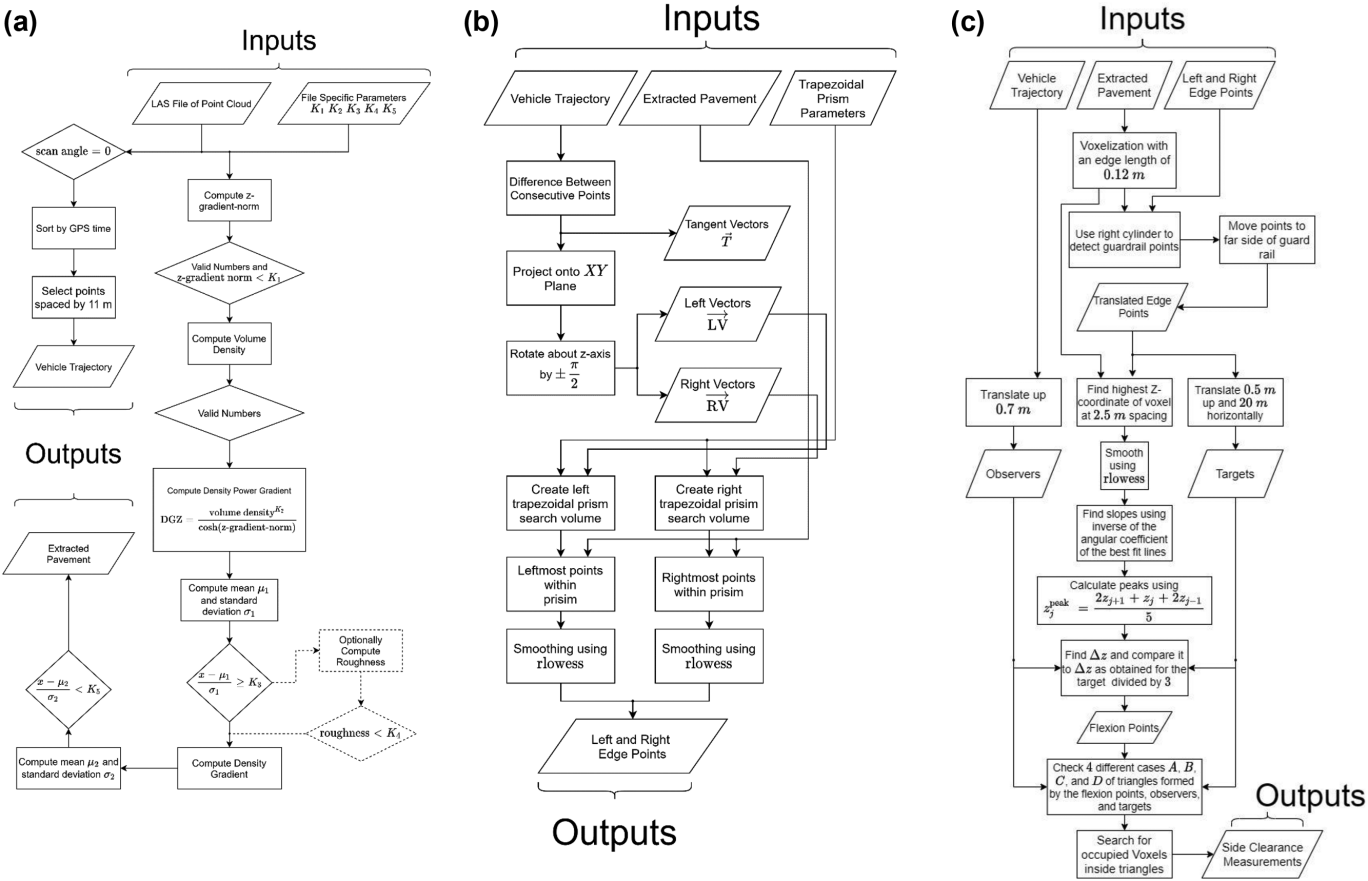
Flowchart of the method.

**Figure 3.**
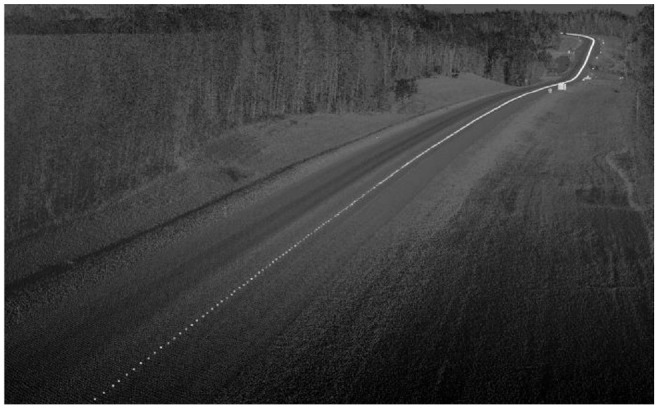
Example of vehicle trajectory points.

Figure 2, *a–c* show a flowchart of the proposed method. First, the scanning vehicle trajectory was extracted from the point cloud. The scanning vehicle trajectory defines a set of points along the travel lane, as shown in Figure 3, which were used in the road edge searching algorithm to determine the edge of the roadway. Pavement surface points were then detected through a series of filtering steps performed on the point cloud. These filters use geometric features, including the maximum z-gradient norm, density power, density power per z-gradient norm deviation, roughness deviation, and density gradient deviations, after which, the edge trajectories of the pavement were extracted by generating a solid shape on either side of the points along the trajectory point set. Points within the shape were determined via a volume comparison by generating pyramids between the point of interest and the faces of the solid shape. Once pavement edges were extracted and smoothed, a guard rail detection was performed using tangent vectors from the road edge and cylindrical proximity to mark the existing safety barriers. The roadside slope was then calculated with a best-fit procedure using the normals of nearby points along the slope.

With the slope analysis complete and the pavement edge defined, a set of observer points was constructed by translating from the pavement edge trajectory. Observer points are perspectives from which the roadside was analyzed. When identifying the regions in which to perform obstacle searches, targets were placed 20 m into the roadside zone, and flexion points were identified. These targets and flexion points together determine the regions searched for obstacles by raycasting, bounding the roadside zone search. Finally, a voxel-based raycasting approach was used to search for obstacles. The regions outlined for searching were determined based on where targets and flexion points were generated. The layout of these regions was one of four different scenarios, for every interval between two observers, which defined the process in which the raycast-voxel detection was performed.

### Edge Trajectory Extraction

To calculate the edge trajectory and extract it from the point cloud, three processes were performed. First, the vehicle trajectory was extracted. The vehicle trajectory provides an ordered sequence of reference points from which the edge searching algorithm was later activated. After vehicle trajectory is extracted, the pavement was extracted from the point cloud based on several geometric features. By extracting the pavement, a much smaller relevant set of points was formed from which the road edge trajectory was identified. This increased the precision of the edge search by pre-emptively excluding points that were not located on or near the extracted pavement.

#### Vehicle Trajectory

The trajectory of the vehicle was created from the point cloud by selecting points with zero scan angle. These points lie directly below the scanner. Selected points were used to create a trajectory by ordering them chronologically and selecting points sequentially, which were at a minimum distance of 11 m from the previous point. Tangents to the trajectory were made by taking the vector difference ( Δx,Δy,Δz) of the following point and the current point. By doing so, a point set P was generated, that defined the points at which the edge search occurred.

#### Pavement Extraction

Given an entire point cloud, this stage was designed to extract only the points located on the pavement of the road. The approach used was based on multiple geometric feature-based filtering steps using heuristically defined parameters. Table 1 shows the values of the geometric features and parameters used. The features are the maximum z-gradient norm ( $K_{1}$ ), density power ( $K_{2}$ ), density power per z-gradient norm deviations $\left(K_{3}\right),$ roughness deviation $\left(K_{4}\right),$ and density gradient deviations $\left(K_{5}\right)$ , as a function of the number of lanes selected. The stages of filtering by roughness deviations and by density gradient were applied only when the respective constant was set. Below is a definition of the variables used in the filtering process (*33*, *34*):

**Table 1.** Values of geometric features and parameters selected for filtering as a function of the number of lanes.

Number of lanes	$K_{1}$	$K_{2}$	$K_{3}$	$K_{4}$	$K_{5}$
1	0.0675	1.3	–1.175	0.925	-
2	0.0675	1.1	–1.15	0.875	–0.005
More	0.065	1.25	–1.225	0.925	-

- Roughness: is equal to the distance between a point and the best fitting plane to its neighbors.
- Gradient: is the rate of change in any feature value at a point (e.g., elevation) compared with its neighbors.
- z-gradient: is the gradient of the elevation of a point relative to its neighbors.
- Number of neighbor density (*N*): counts the number of neighbor points to each point in the point cloud within a sphere of radius *R*.

All values with a z-gradient norm greater than or equal to $\left(K_{1}\right)$ were filtered out. Once the first generic filtering stage was performed, point cloud volume density was calculated for a radius equal to 0.45 m, the roughness was calculated for a radius equal to 1.0 m, and the density gradient norms were also calculated when this option was applied.

The density power per z-gradient norm ( DZG ) deviations filter was then applied on the points, the DZG property was defined for each point i as Equation 1 shows. The value was calculated for each point to compute the average ( $\bar{\mathrm{DZG}}$ ) and the standard deviation ( σ(DZG) ) of DZG . Then, all points i satisfying Equation 2 were filtered out.

(1) $$\begin{array}{c} {\mathrm{DZG}}_{i}=\frac{{\mathrm{Density}}_{i}^{K_{2}}}{\cosh\|\vec{\;\nabla }z_{i}\|} \end{array}$$

(2) $$\begin{array}{c} {\mathrm{DZG}}_{i}<\bar{\mathrm{DZG}}+K_{3}\cdot \sigma \left(\mathrm{DZG}\right) \end{array}$$

Two more filters were applied using the same notation of the standard deviation for a variable applied previously. All points with roughness greater than or equal to $K_{4}\cdot \sigma \left(\mathrm{roughness}\right)$ were also filtered out. For the last possible filter to be applied, when the density gradient ( $\vec{\;\nabla }\mathrm{density}$ ) deviations were used, all points satisfying Equation 3 were removed. Therefore, all the remaining points were located on the pavement.

(3) $$\begin{array}{c} {\left\|\vec{\;\nabla }\mathrm{density}\right\|}_{i}\geq \bar{\left\|\vec{\;\nabla }\mathrm{density}\right\|}+K_{5}\cdot \sigma \left(\|\vec{\;\nabla }\mathrm{density}\|\right) \end{array}$$

#### Edge Search and Smoothing

To use the pavement extracted by the last stage, first, some definitions were made. Normal vectors to the trajectory were calculated for both sides of the scanning direction (left and right) by taking the tangent vector with no z component ( Δx , Δy , 0) and rotating it about the z-axis counter-clockwise by $\frac{\pi }{2}$ or $-\frac{\pi }{2}$ for left and right, respectively.

**Figure 4.**
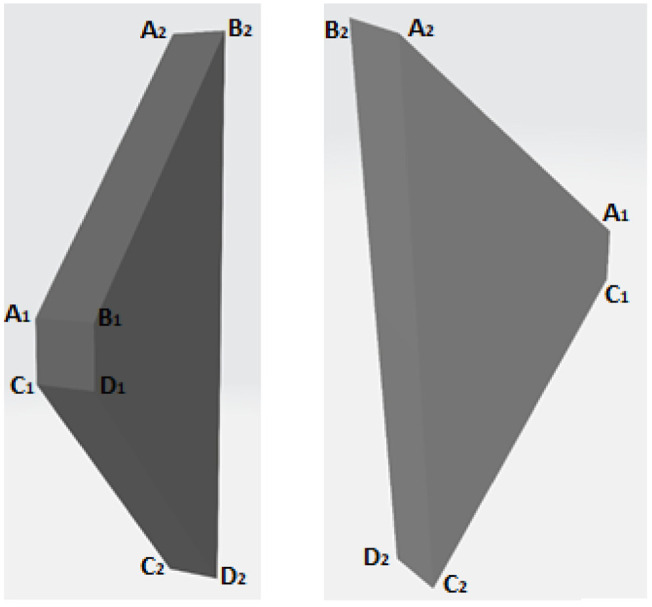
Two different views of the solid used for the search of candidate points to be on the edge of the road.

As part of the proposed procedure to find the candidate points to be on the edge of the road, a solid used for the search was defined as shown in Figure 4. If a point was within the solid, it was considered a candidate point. To define the solid, some additional constants were introduced.

First, the initial z -window ( ZW=0.1m ) and the z -step ( ZS=0.4 ) were defined. The first one was the length of the shortest parallel side of the trapezoid defining the solid, while the second was half the slope of the inclined faces to the horizontal planes. Then, the last parameter defined and used for both right and left sides was the half solid depth ( $\mathrm{HSD}=\frac{4}{3}m$ ). This value can be visualized in Figure 4 as half of the thickness through which the trapezoid was translated.

To fully define the shape, the last parameter needed was the height of the trapezoidal faces which was different for the left and right sizes. For the right side, it was defined as the right solid width ( RSW ) shown in Equation 4 and was calculated as a function of the lane width ( LW=4m ), the number of lanes in each direction ( LN ), and the car lane number ( CL ) from 1, the leftmost to LN , the rightmost.

(4) $$\begin{array}{c} \mathrm{RSW}=5+\mathrm{LW}\cdot \left(\mathrm{LN}-\mathrm{CL}\right) \end{array}$$

Using all measures described, Equation 6 shows how the solid volume for the right side ( RSV ) could be calculated. Similarly, Equation 7 shows the solid volume for the left side ( LSV ) as a function of the left solid width ( LSW ) defined by Equation 5.

(5) $$\mathrm{LSW}=\{\begin{array}{c} 5+\mathrm{LW}.(\mathrm{LN}+\mathrm{CL}-1),\mathrm{if}\;\mathrm{two}\;\mathrm{way}\;\mathrm{road}\\ 5+\mathrm{LW}.(\mathrm{LN}-1),\mathrm{if}\;\mathrm{one}\;\mathrm{way}\;\mathrm{road} \end{array}$$

(6) $$\begin{array}{c} \mathrm{RSV}=2\cdot \mathrm{HSD}\cdot \left(\mathrm{ZW}\cdot \mathrm{RSW}+\left(\frac{\mathrm{ZS}}{2}\right)\cdot \mathrm{RS}W^{2}\right) \end{array}$$

(7) $$\begin{array}{c} \mathrm{LSV}=2\cdot \mathrm{HSD}\cdot \left(\mathrm{ZW}\cdot \mathrm{LSW}+\left(\frac{\mathrm{ZS}}{2}\right)\cdot \mathrm{LS}W^{2}\right) \end{array}$$

To find out if a point was within the solid, the volumes of the pyramids formed by the point and each of the faces of the solid were calculated. If the sum of these volumes was less than or equal to the volume of the solid, the point was inside or on the surface of the solid. This process is described hereafter. To use the solid in the search, first, its vertices ( $A_{1}$ , $B_{1}$ , $C_{1}$ , $D_{1}$ , $A_{2}$ , $B_{2}$ , $C_{2}$ , and $D_{2}$ ) were defined, and then the faces x were found. The procedure was done for each point $P_{i}$ with its corresponding normalized tangent vector $\vec{T_{i}}$ , and normal vectors $\vec{RV_{i}}$ , on the right, and $\vec{LV_{i}}$ , on the left, in the scanning vehicle’s trajectory. Equations 8 and 9 show how the vertices were defined for the right and the left sides, respectively. Finally, Equation 10 shows how the faces were defined using the vertices.

(8) $$\begin{array}{c} A_{1}=P_{i}-\mathrm{HSD}\cdot \vec{T_{i}}-\left(\frac{\mathrm{ZW}}{2}\right)\cdot \vec{z}\\ B_{1}=A_{1}+\mathrm{ZW}\cdot \vec{z}\\ C_{1}=B_{1}+2\cdot \mathrm{HSD}\cdot \vec{T_{i}}\\ D_{1}=C_{1}-\mathrm{ZW}\cdot \vec{z}\\ \begin{array}{c} A_{2}=A_{1}+\mathrm{RSW}\cdot \left(\vec{RV_{i}}-\left(\frac{\mathrm{ZS}}{2}\right)\cdot \vec{z}\right) \end{array}\\ B_{2}=A_{2}+\left(\mathrm{ZW}+\mathrm{ZS}\cdot \mathrm{RSW}\right)\cdot \vec{z}\\ C_{2}=B_{2}+2\cdot \mathrm{HSD}\cdot \vec{T_{i}}\\ D_{2}=C_{2}-\left(\mathrm{ZW}+\mathrm{ZS}\cdot \mathrm{RSW}\right)\cdot \vec{z} \end{array}$$

(9) $$\begin{array}{c} A_{1}=P_{i}-\mathrm{HSD}\cdot \vec{T_{i}}-\left(\frac{\mathrm{ZW}}{2}\right)\cdot \vec{z}\\ B_{1}=A_{1}+\mathrm{ZW}\cdot \vec{z}\\ C_{1}=B_{1}+2\cdot \mathrm{HSD}\cdot \vec{T_{i}}\\ D_{1}=C_{1}-\mathrm{ZW}\cdot \vec{z}\\ \begin{array}{c} A_{2}=A_{1}+\mathrm{LSW}\cdot \left(\vec{LV_{i}}-\left(\frac{\mathrm{ZS}}{2}\right)\cdot \vec{z}\right) \end{array}\\ B_{2}=A_{2}+\left(\mathrm{ZW}+\mathrm{ZS}\cdot \mathrm{LSW}\right)\cdot \vec{z}\\ C_{2}=B_{2}+2\cdot \mathrm{HSD}\cdot \vec{T_{i}}\\ D_{2}=C_{2}-\left(\mathrm{ZW}+\mathrm{ZS}\cdot \mathrm{LSW}\right)\cdot \vec{z} \end{array}$$

(10) $$\begin{array}{c} \begin{array}{c} F_{1}=\left(A_{1},B_{1},C_{1},D_{1}\right)\\ F_{2}=\left(A_{2},B_{2},C_{2},D_{2}\right)\\ F_{3}=\left(A_{1},B_{1},A_{2},B_{2}\right)\\ F_{4}=\left(C_{1},D_{1},C_{2},D_{2}\right)\\ F_{5}=\left(B_{1},C_{1},B_{2},C_{2}\right)\\ F_{6}=\left(A_{1},D_{1},A_{2},D_{2}\right) \end{array} \end{array}$$

For both sides, the area $S_{f}$ of all faces and the normalized normal vector to the surface $\vec{N_{f}}$ were calculated. To reduce the processing time of the search, for each side, a rough bounding box was defined and all points outside the bounding boxes were filtered out. To define the remaining points on the right side ( PR ) and the equivalent set of points for the left side ( PL ), every point was tested to determine whether it was inside the solid of its corresponding side.

For each point $P_{j}$ , the volume $VP_{i}$ of a pyramid created by each of the six faces of the solid and the point was calculated using the formula $VP_{i}=\left(1/3\right)\cdot \mathrm{base}\cdot \mathrm{height}$ . The total volume for a point was the sum for the six faces: $VP_{j}=\sum_{i=1}^{6}VP_{i}.$

Afterwards, $VP_{j}$ was analyzed to determine if the point was inside or in the surface of the solid by comparing it with RSV (for the right side) or LSV (for the left side). If the volume found was greater than the volume of the search solid for the respective side, it was considered outside the solid; otherwise, it was inside. For each of the points inside each solid, the point furthest from the trajectory point was stored as the left edge point and right edge point.

As the set of points selected can be noisy, a smoothing stage was applied to the data. First, for each point $P_{i}$ in the trajectory, the vector from this point to the edge points ( ${\vec{\mathrm{REV}}}_{i}$ , for the right side, and ${\vec{\mathrm{LEV}}}_{i}$ , for the left side) was calculated as the difference between the right or the left edge i -point and $P_{i}$ . Then, for each one of the vectors calculated before, the Euclidian norms and unitary vectors ( ${\vec{\mathrm{rev}}}_{i}$ or ${\vec{\mathrm{lev}}}_{i}$ ) were found.

The Euclidian norms calculated were the distances from the trajectory to the edges and were the desired variables to be smoothed. MATLAB’s smooth function was used to smooth the norms using the ‘rlowess’ method, which is robust against outliers. This resulted in a set of smoothed right or left distances ( $\mathrm{SR}D_{i}$ and $\mathrm{SL}D_{i}$ ).

Finally, the final edge trajectory points ( $RE_{i}$ and $LE_{i}$ ) were obtained by applying the smoothed distance to the unitary vectors calculated before, as shown in Equation 11. To keep the same format used before to trajectory points, the edge trajectory vectors from each point to the subsequent one were calculated and stored to format the output given.

(11) $$\begin{array}{c} \begin{array}{c} RE_{i}=P_{i}+\mathrm{SR}D_{i}\cdot {\vec{\mathrm{rev}}}_{i}\\ LE_{i}=P_{i}+\mathrm{SL}D_{i}\cdot {\vec{\mathrm{lev}}}_{i} \end{array} \end{array}$$

### Checking for Guard Rails

It was necessary to check for guardrail presence on the road, as sometimes the pavement extends beyond them, consequently placing the edge trajectory outside of the guardrail. For that reason, when there was a guard rail on the edge of the road, the observer points were placed on the close side of the guardrail so that it was detected later when checking for obstacles. This process starts with the translation of the edge points up by the defined guardrail height of 0.30 m and then the normal vectors were calculated for edge points in a similar way to the original trajectory; calculating tangents as a difference between two points and then rotating them about the z-axis $\pm \frac{\pi }{2}$ . The normal vectors were oriented, facing away from the road.

All the vectors and the edge points were voxelated to improve performance when searching for the guard rails. Details about voxelization may be found in (*18*). Each voxel is a cube with a side-length of 0.12 m. After the normal vectors and the edge point’s vectors were normalized, the voxels and the vectors created were used to search for obstacles. Basically, for each edge trajectory point, a search was done for points inside an inclined cylinder facing the road.

The inclination was dependent on the z− bias configured as 0.15 voxels, the radius of check used was 0.1 m and the height of the cylinder was configured as 0.3 m for the right side and 0.4 m for the left side.

If an obstacle was found, the edge trajectory point was placed on the side of the obstacle that was closest to the original trajectory array. In the end, all points were converted back to the original coordinate system.

### Finding Slopes

To find the slopes on each side of the road, the normal vectors with no z component were calculated and normalized for both edges as done for checking for guard rails. Then, for each side of the road, the highest z values of voxels with sampling step distance (equal to 0.2 m) from each other were taken starting with 1 m from behind the edge trajectory to 20 m from the trajectory, following the normal vector calculated before.

The approach described can be done from an interval of length equal to two times the height of the search (configured as equal to 17.5 m) in which the z value of the trajectory point was centered. For this interval, points were searched inside a radius of search configured as 0.5 m by default. The procedure adopted was necessary to allow the smoothing of outliers at the start of the sampling to be more predictable.

Later, the z values taken were smoothed using the same weighted local regression method used to smooth the edge points, considering 7 to be the number of neighbors. All the smoothed points behind the trajectory of the edges were removed, and the remaining points were separated in intervals of 2.5 m.

For each interval created, if there were at least two points inside the interval, the best-fit line was calculated for them to find the slope, typically equal to the inverse of the angular coefficient. The expected change in height for the fitting length interval was also calculated.

**Figure 5.**
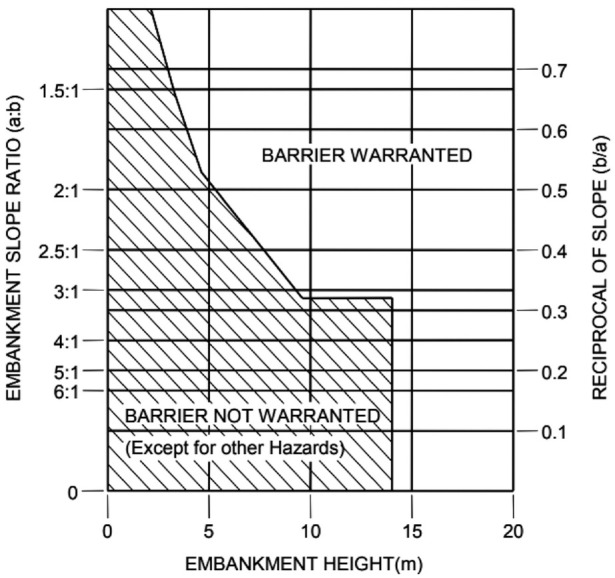
Warrant criteria for barrier requirements.

All the slopes evaluated were classified following the road standards and compared. To analyze the change in height, the values were accumulated for each point and their values were classified to evaluate if a barrier was warranted or not according to (*4*) as shown in Figure 5.

### Targets, Observers, and Flexion Points

Observer points were defined as the edge trajectory points for each side after they were leveled to the ground and the leveling then fixed by checking for “peaks” in the height and translating them up by 0.7 m. Targets were the farthest points used in the search for obstacles at the roadside. The targets were considered as points following the direction of the edge’s trajectory normal vectors shifted 20 m horizontally and 0.5 m up from the ground. While leveling the targets to the ground, if the ground at the target position was too high, flexion points were generated. The flexion points have the purpose of adapting the search area to the ground surface of the roadside.

#### Leveling Points to Ground

First, for each point to be leveled, the minimum and maximum z coordinates to search for the ground were calculated by the point’s z coordinate shifted down and up 20 m respectively. Then, the normal vectors to the path with no z component were calculated as done before for the edge’s trajectory, and the points and vectors were converted to the voxel coordinates.

**Figure 6.**
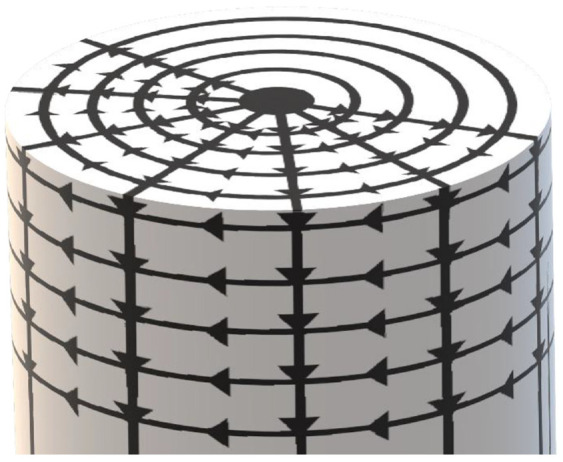
Cylinder grid used to search for the ground surface.

After the initial process described, a search for the highest z value of the closest occupied voxel was executed for each point to be leveled. This search was done in a cylinder around the point and between the z values calculated before. Figure 6 shows how the cylinder was searched, top-down, and outwards. This was done with voxel coordinates using steps of 0.999 voxels to ensure that no voxels were skipped in the search process. Then, for the selected point, its height was set as the highest z value found minus the minimum z coordinate acceptable. The difference of height between the original and the modified point ( Δz ) was registered and used to find flexion points.

To smooth the data after the leveling process, a search for peaks was made, and if a peak was found, its z coordinate was changed for the weighted average between its coordinate and its neighbors’ coordinates, as shown in Equation 12 for a point j considered as a peak.

(12) $$\begin{array}{c} z_{j}^{\mathrm{peak}}=\frac{2\cdot z_{j+1}+z_{j}+2\cdot z_{j-1}}{5} \end{array}$$

To figure out if the point $P_{j}$ was a peak, first, the difference in height between it and its previous point was calculated and divided by the distance between these two points. Then, the same operation was repeated using the selected point and its successor, resulting in two different ratios. If the mean value of these two ratios was greater than or equal to a threshold value configured to be 0.375, $P_{j}$ was considered as a peak and smoothed by Equation 12. This was necessary to avoid obstacles being used as the highest point for the roadside ground.

Next, the flexion points were calculated. The first task of this activity was to check for each point if the selected one had a Δz greater than or equal to 1.2 m. Then, for the current point, a search was done to find the highest z value, closer to the road, in the vicinity of the point using the same maximum and minimum parameters of the leveling process. If a value was found, the $\Delta z_{\mathrm{new}}$ from this highest z value to the current observer was calculated and compared with the Δz obtained for the target divided by 3.0 $(\frac{\Delta z}{3}$ was used as the $\Delta z_{\mathrm{new}}$ to be accepted as a flexion point). It is important to note that flexion points were not a fixed distance from either the target or the observer, their distance was dependant on the roadside surface.

### Side Clearance Measurement

With all generated targets, observers, flexion points, and vectors converted to the voxel world, an obstacle detection was made for both sides of the road. Basically, for each target-observer pair inside a region, the existence of flexion points associated with them was checked to decide what type of region check would be applied. The region check applied returned points in which an obstacle was found and calculated the distance from the trajectory, which was the side clearance at that point in the trajectory.

#### Region Check

**Figure 7.**
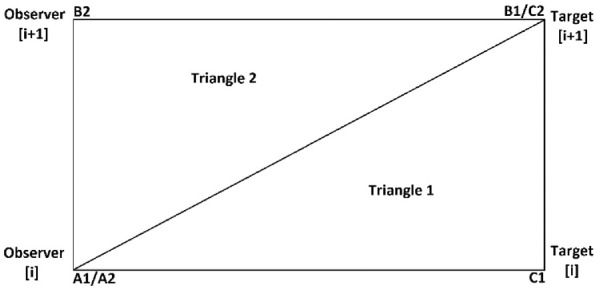
The triangles defined using the pairs of observer-target to do a region check for case A.

There were four types of regions defined to be checked depending on the relations between the observer-target pair selected and possible flexion points. For the first possible case (type A), if both the current observer-target pair and the next observer-target pair did not have flexion points associated with them, the triangles to be checked were defined as shown in Figure 7.

**Figure 8.**
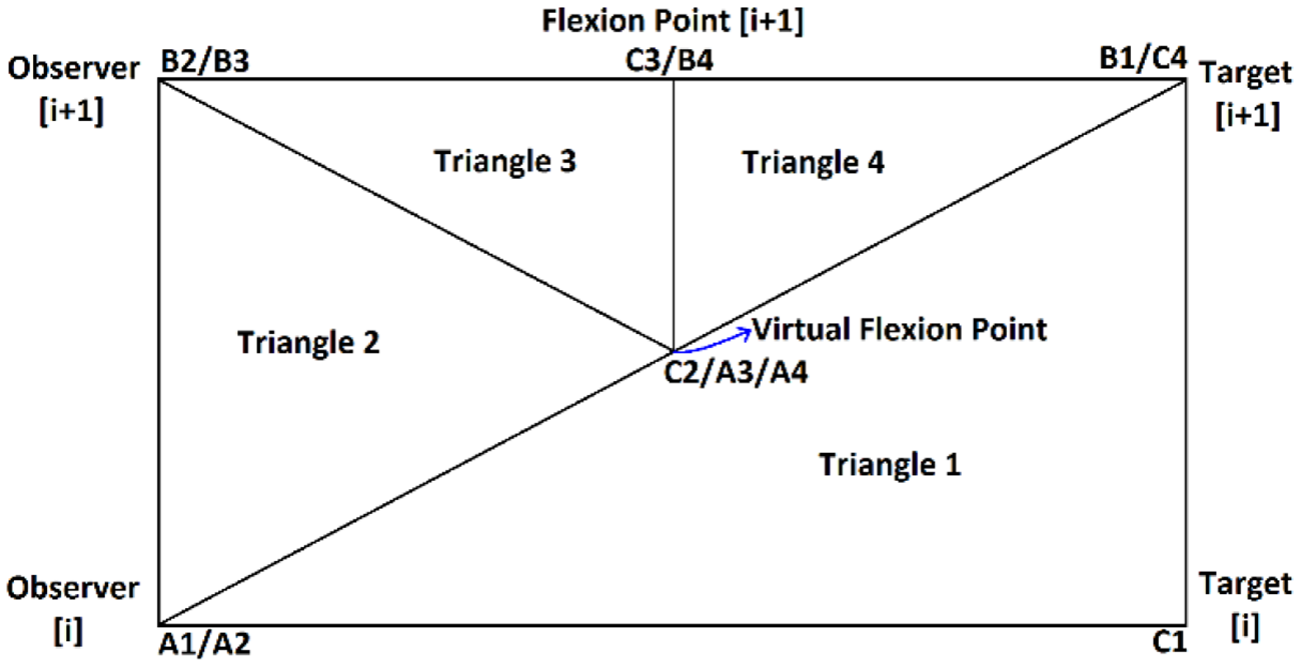
The triangles defined using the pairs of observer-target to do a region check for case B.

The next possible case (type B) was defined for observer-target pairs that did not have a flexion point associated with them, but the next one had. In this situation, two more triangles were defined, resulting in the region shown in Figure 8.

**Figure 9.**
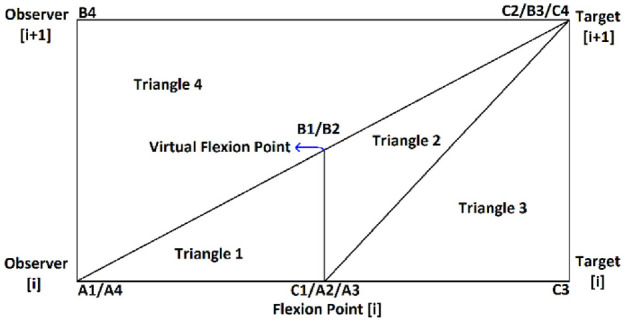
The triangles defined using the pairs of observer-target to do a region check for case C.

For the situations in which the opposite behavior in relation to type B was found, there was a new case (type C) defined, and the four triangles to check were defined as can be seen in Figure 9.

**Figure 10.**
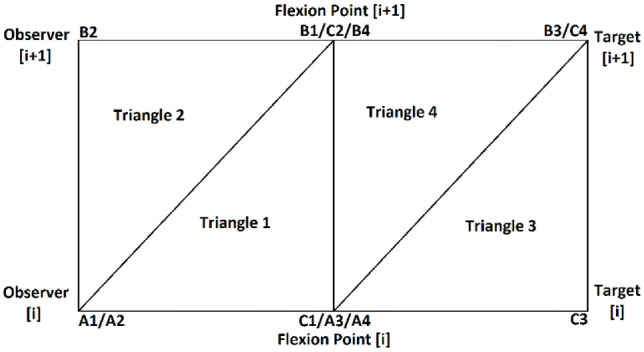
The triangles defined using the pairs of observer-target to do a region check for case D.

Finally, the last possible case (type D) occurred when both the selected observer-target pair and the next one had flexion points associated with them. For this situation, the triangles defined can be seen in Figure 10.

For all cases, each triangle was analyzed individually. In Figures 7–10, each triangle i was defined by the sequence of vertices $A_{i}$ , $B_{i}$ , and $C_{i}$ . So, the vectors $\vec{A_{i}B_{i}}$ and $\vec{A_{i}C_{i}}$ could be calculated for every triangle and then normalized. Then, a search for an occupied voxel was done for the points inside the triangle i by varying the value of the $\vec{A_{i}B_{i}}$ , calculating the respective $\vec{A_{i}C_{i}}$ , and searching between them. If an occupied voxel was found, the point was considered an obstacle, and its position and distance from the trajectory were registered and used to build the side clearance measure.

### Validation

Autodesk ReCap, Civil 3D, and CloudCompare were used to validate the output obtained from the method (*33*, *35*, *36*). To validate the existence of obstacles, the point cloud data and the location in Google Street View were manually reviewed to identify obstacles’ types and locations (e.g., poles, trees, etc.) and compared with the output from the proposed method. Several parameters, such as the values of the geometric features, were optimized using one of the test segments or designed based on logic conditions that may exist in a rural environment. For instance, the parameters used to extract the pavement were determined using one test segment. Detected obstacles were visually verified using Google Earth street view images. The distance to an obstacle was manually validated in CloudCompare by selecting a point on the edge and a point on the obstacle. For pavement edges validation, the .LAS file was first converted to a .RCP file using ReCap. The resulting .RCP file was inserted into Civil 3-D as a point cloud attachment to maintain point cloud density. Then, the edge line was drawn manually and compared with the model result.

For accuracy, the calculation is based on the goodness-of-fit of the model-generated edge to the manually extracted edge (ground truth). First, the coordinates of extracted points were transferred to coordinates on the road. For every point, its distance from the beginning of the road and distance from the vehicle’s trajectory were calculated. Then, the displacement between points on the model-generated edge and the ground truth was calculated. Finally, the ratio between the displacement and the distance of the ground truth is used as a measure of accuracy.

Finally, the slope data were validated. The first step was to import the point cloud data into CloudCompare and have it overlapped by a grid of points representing the exact points generated by the algorithm. Through the use of CloudCompare’s tools and an estimation of a surface given by the points and their neighbors, each point had its normal calculated. From that, a few arithmetic steps are applied to calculate the grade at every point in the point cloud. This information about grades was overlapped onto the point cloud. Finally, a comparison between the grades obtained from the model and the grades from CloudCompare was performed. The output of the two methods was almost identical except for regions with heavy grass. This is because the CloudCompare method does not operate solely on the highest points but instead takes all points within a sphere around the point into account. Several attempts using ground removal algorithms failed to remove the grass points. Regardless, this method validated the calculations of the proposed algorithm.

## Results and Discussion

**Figure 11.**
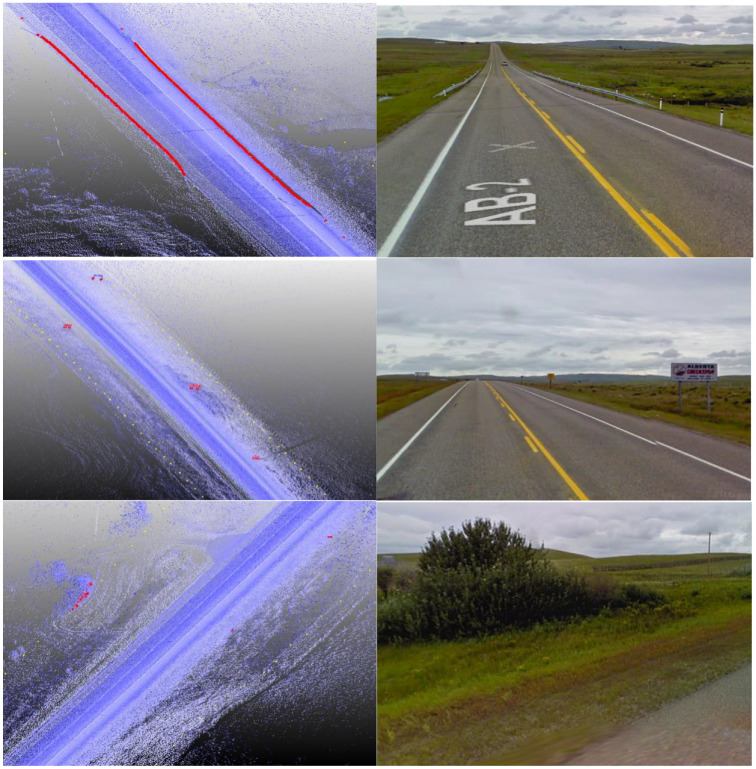
Guardrails, signs, and vegetation detected on Highway 2.

**Figure 12.**
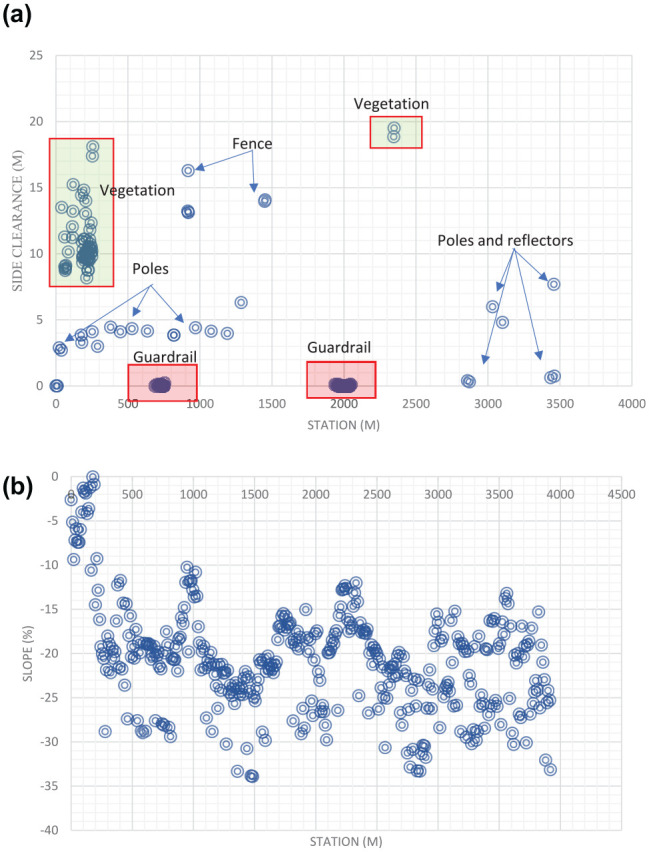
Graphs on the right roadside of Highway 2: (*a*) side clearance as a function of the station (m); and (*b*) slope (%) of the roadside as a function of the station.

**Figure 13.**
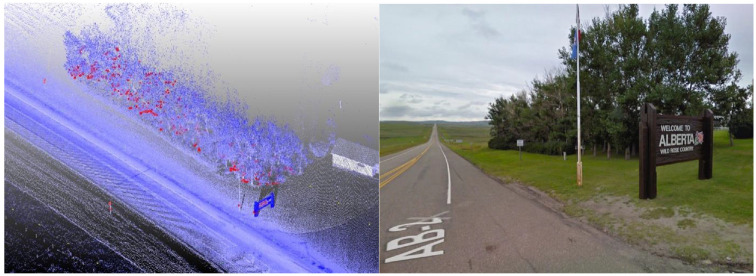
Detections at Carway exit / Alberta entrance region.

**Figure 14.**
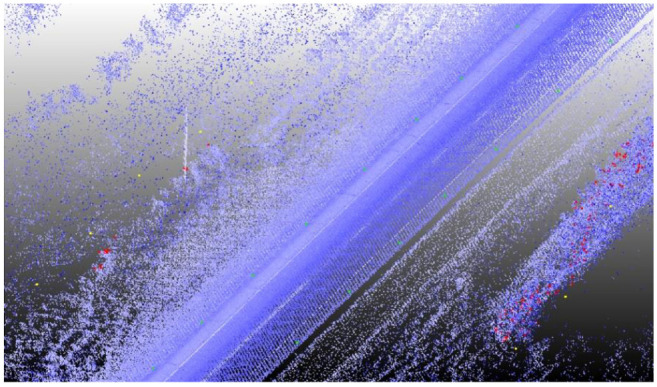
Sample detections on Highway 63.

**Figure 15.**
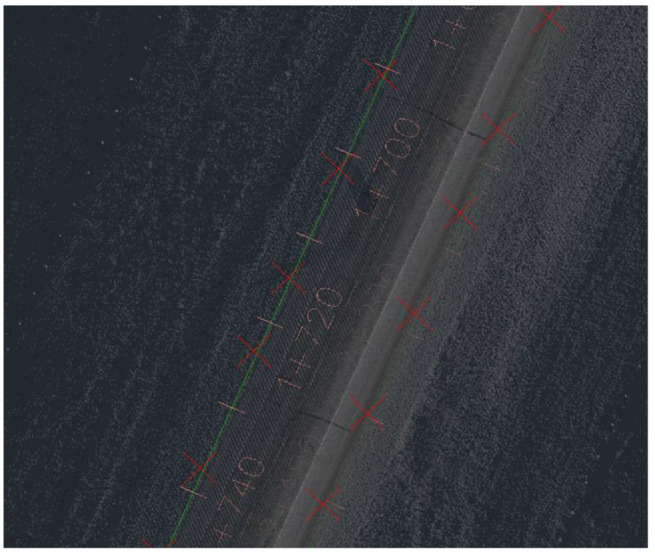
Edge comparison in Civil 3-D and Autodesk ReCap.

Figure 11 shows samples of the analysis results on highway 2. In Figure 11, the green points represent edge trajectory observer points, yellow points show the targets used in region checks, and red points show all detected roadside features. Figure 11, row 1 left, shows an instance where right and left roadside guardrails and reflectors were detected in the point cloud. Figure 11, row 1 right is a Google Street View of the same location. Figure 11, row 2 shows detected sign poles on the same segment. Figure 11, row 3 are sample detections of roadside vegetation in the same segment. Figure 12 shows the mapped side clearance and roadside slopes on the right side of the scanning vehicle on the Highway 2 segment. Red shaded areas show locations of guardrails on the graph with a side clearance of 0 m at stations (0+704 to 0+750) and (1+952 to 2+042). The lengths of the right side of the two guardrails were 46 m and 90 m. Figure 12 shows the 90 m guardrail starting at station 1+950. The blue shade shows a suburban area at the beginning of the test segment. The noisy area with low side clearance and small ground slopes is on the edge of the suburban region of Carway on the US–Canada border. The roadside features detected were trees, light poles, and sign poles, as presented in Figure 13. It is important to note that obstacles that fall outside of the analysis zone (e.g., the building in Figure 13) are not detected in the analysis. The green shaded area on the graph shows the locations of trees. The different groups of points were labeled with what they represent. Figure 14 shows a sample of the output on Highway 63. Most detections in this segment were related to electric poles or trees. Figure 15 shows a sample of the manually created edges (green line) using Civil 3-D and Autodesk ReCap and the edge points created by the proposed method (red X-shaped points). The accuracy of edge detection ranged from 97% to 98.5%.

Figure 12 shows two scatter plots, (*a*) and (*b*). The former depicts side clearance as a function of the station (m), and the latter depicts the slope (%) of the roadside as a function of the station. The station is defined as the distance along the segment. The side clearance plot, (*a*), highlights and labels obstructions based on the obstruction type. The first highlighted section contains detected vegetation near the Carway exit, shown in Figure 13, ranging from around 8 m to 18 m from the roadside. The next labeled points were poles/signs, which appear to be sparsely positioned but range in station more than in clearance from the roadside, as signs normally exist near the roadside. The next highlighted points were detections of guard rails, which are dense and positioned right along the roadside. Fencing is the next category of obstructions, which are somewhat dense and can range in either clearance or station depending on their location. Lastly, a pattern of poles and reflectors is seen. It should be noted that reflectors have a similar pattern to other signs and poles with sparse positioning ranging more in station than clearance, but are different in that they exist close to the edge of the roadway, similar to guard rails.

The second plot, showing the slope as a percentage, complements the first, as it was measured over the same segment station such that the obstructions in (*a*) at some station are on a slope given by (*b*) at the same station. The slope ranges from about 0% to −34%. Critical slope conditions, as an absolute value, are those that increase the chance of vehicle rollover which is considered greater than 33.3% (*4*). Slopes below 25% are considered traversable and thus have a recommended clear zone width ranging from 2 m to 15 m depending on the road speed and average annual daily traffic (AADT) (*4*). Those that breach 25% slope and do not meet the recommended clear zone width should be cleared of obstacles for a compensation distance at the toe of the slope. The same goes for any clear zone that breaches a critical slope, >33%. As evident in this section, calculating clearance and slope as a function of station may allow the types of obstructions to be predicted based on patterns of positioning. Additionally, it can also identify locations that breach the given roadside design guidelines. The tool also extracts embankment heights and automatically generates a list of all locations where a guardrail is warranted.

**Figure 16.**
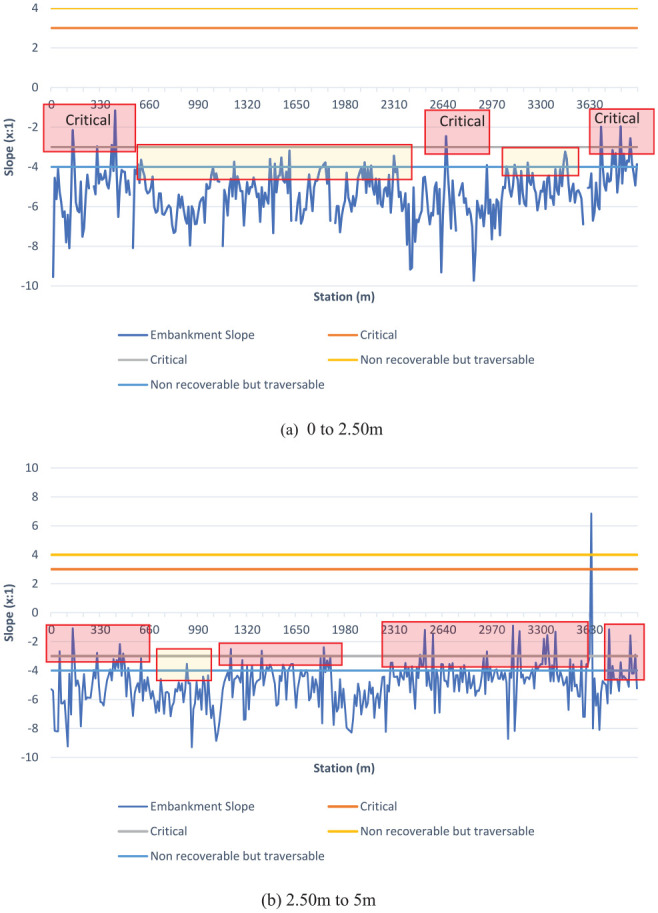
Slopes to the left side of Highway 2 Segment 2: (*a*) the 0 to 2.5-m strip; and (*b*) the 2.50-m to 5-m strip.

**Figure 17.**
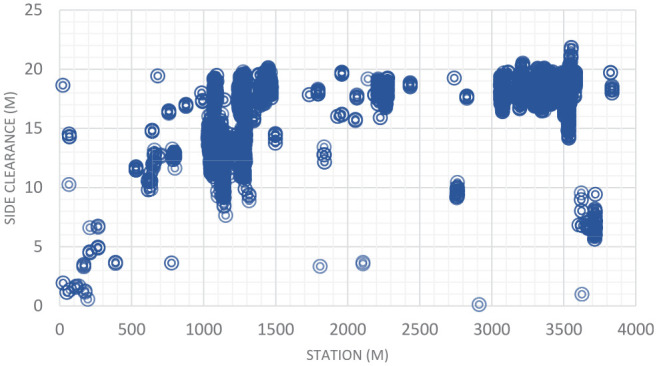
Side clearance on Highway 2 Segment 2.

As explained above, slopes were extracted within a grid of points on the roadside. The spacing of the grid points used in this example was 2.50 m in the perpendicular direction to the road. Figure 16 shows the output of the slope on the two 2.50 m strips adjacent to the road edge on the left side of the scanner on Highway 2 segment 2. Slopes are presented in the (XH:1V) format, which is horizontal to vertical ratio, commonly used in design guidelines (*2*–*4*). A comparison at several locations with a calculation procedure in Cloud Compare (*33*), yielded similar results. The tool automatically classifies slopes according to Alberta design guidelines classes, critical (steeper than 3:1), non-recoverable but traversable (between 4:1 and 3:1), and recoverable (flatter than 4:1). In addition, embankment heights were recorded for each 2.50-m step. As evident from Figure 16*a*, some locations (shaded red) breach the critical steepness, and numerous locations breach non-recoverable steepness. Side clearance results are presented in Figure 17. As evident from the combination of these graphs, some locations with critical slopes have fixed objects present on the roadside, which is against the guideline recommendation. It is worth mentioning that Alberta design guidelines define clear zone dimensions based on traffic volume and road design speed. Therefore, the method can be used to automatically verify compliance with guidelines and identify locations that require further assessment and review.

Tall grass can become an obstacle and impact the performance of the proposed and traditional methods. Roadside mapping and assessment are typically coordinated with the provincial vegetation management program (PVMP) in Alberta (*37*, *38*). As such, the scanning process can also be coordinated with a PVMP. In PVMP, mowing activities are performed on highways to keep the weed height to a minimum. This is essential to improve the road and roadside visibility, to ensure the safety of travelers and rural communities, to ensure proper drainage to avoid damage to the road infrastructure, and to minimize wildfires risk, which is a major concern in Alberta. For instance, in Strathcona County, Alberta, the highway right-of-way is typically mowed twice a year. Spraying herbicide is another tool used to prevent weeds from growing and spreading to the road. As such, the scanning can be performed right after mowing activities are carried out. In this case, the fast data collection by lidar (as opposed to manual collection and assessment) can allow large-scale data collection before the flat/smooth weed surface grows (*37*, *38*).

If scanning cannot be done on the mowed roadside, one could seemingly use the grass surface as an approximation of the ground. This approximation is based on the assumption that the grass’s height is normally distributed, backed up by Gibb et al. (*39*), who showed that both frequently and infrequently grazed swards exhibit normal distributions in height. This assumption allows for regression to be done on the grass height, from which an estimation of roadside slope can theoretically be obtained.

With the method proposed in this study, noncompliance with roadside design guidelines (*4*) can be automatically identified on a large scale. This information may be provided to transportation agencies to further assess these locations and determine any actions to take, including non-action, that is, justification of the guideline violation. In doing so, transportation agencies can optimize the use of limited resources traditionally allocated to manual documentation of roadside clearance parameters. Furthermore, the collected information can be used to study the safety performance of existing designs using crash data and to develop performance-based design guidelines.

## Conclusions

In this study, a novel voxel-based raycasting approach is proposed for automating the roadside mapping and assessment process and for collecting roadside clearance data (distance to obstacles, slopes, and embankment height). The method was tested on four highway segments in Alberta, Canada. This topic has not been explored in previous research, as the target in most previous studies was the detection of a certain element of the road without a focus on automated roadside mapping or assessment. MLS data were collected using a scanning vehicle equipped with a RIEGL VMX 450 system. First, the trajectory of the scanning vehicle was extracted from the data via identifiable points with a scan angle of 0 ° . Pavement surface points were then detected using several point-based filtering techniques based on density, roughness, and z-gradient. Once the pavement surface was detected, a specifically designed approach was used to extract the pavement edge trajectories. After the pavement edges were extracted and smoothed, observer points were placed at a certain height above the detected edge trajectory points as perspectives from which the roadside was analyzed. A vertical search algorithm was used to detect the ground surface and place targets/observers above the ground while smoothing peaks. Target points were placed 20 m into the roadside zone, perpendicular to the road, and a certain height above the ground. As pavement edge points may be located after a guardrail, a conical frustum was used to search for guardrail-like obstacles and place the points back toward the vehicle trajectory. This was to detect guardrails as obstacles as the final process of the method involves raycasting between observers, targets, and flexion points in calculated patterns to search roadside regions for obstructions. Additionally, this method also involves calculating the embankment heights and roadside slopes at intervals using a smoothing and best-fit line approach. Finally, design guideline requirements (*3*, *4*) were automatically checked for compliance, and substandard locations were identified. The method showed highly accurate detection and mapping performance on all the tested segments, being successful in detecting all obstacles.

The proposed method can help transportation authorities map and inventory roadside clearance parameters (e.g., distance from features to the road edge, grades, and embankment heights) on a large scale. By processing highway scans with this method, locations that do not meet design guidelines can be automatically flagged for assessment. Such assessment may decide whether to justify the noncompliance with design guidelines or commission a redesign of the location. In addition, the collected information can be mapped with road collisions in geographic information system software to further study the relationship between crashes and existing roadside parameters. The collision analysis can also include other road design features such as sight distances and horizontal curves information (*40–42*). As such, the process can help develop performance-based design guidelines for roadside clear zones and support decision making on road maintenance and upgrades.

The proposed approach detects hazardous roadside locations and obstacles. As such, it can help follow mitigation strategies recommended in design guidelines (e.g., remove the hazard, redesign hazard, etc.) (*4*). Future studies may explore using a deep learning approach to classify obstacles detected. Besides, testing the method on different road types in mountainous areas is worth further investigation. It is worth mentioning that tall grass can become an obstacle and affect the performance of both the proposed and traditional methods. Therefore, roadside mapping and assessment are typically coordinated with a provincial/state vegetation management program (PVMP) (*37*, *38*). As such, the scanning process can also be coordinated with a PVMP. In PVMP, mowing activities are performed on highways to keep the grass height to a minimum. Thus, the scanning can be performed right after mowing activities are carried out. In this case, fast data collection by lidar (as opposed to manual collection and assessment) can allow large-scale data collection before the flat/smooth weed surface grows (*37*, *38*). Finally, the highly detailed mapped information by this method can help assess/improve roadside design for autonomous vehicles (*18, 21*).
